# Microwave Pretreatment and Enzymolysis Optimization of the Lotus Seed Protein

**DOI:** 10.3390/bioengineering6020028

**Published:** 2019-03-27

**Authors:** Bi Foua Claude Alain Gohi, Jinze Du, Hong-Yan Zeng, Xiao-ju Cao, Kai min Zou

**Affiliations:** 1 Biotechnology Institute, College of Chemical Engineering, Xiangtan University, Xiangtan 411105, Hunan, China; gohibifouaca@smail.xtu.edu.cn (B.F.C.A.G.); tjdujinze@163.com (J.D.); 15773228967@163.com (X.-j.C.); Kaiminzou1146349876@aliyun.com (K.m.Z.); 2 Biology and Chemical Engineering School, Panzhihua University, Panzhihua 617000, Sichuan, China

**Keywords:** microwave, lotus seed protein, irradiation powers, structural changes, response surface method, hydrolysis degree

## Abstract

Pretreatment with a microwave was conducted before enzymolysis and shown to enhance the enzymolysis, which changed the secondary structure of the lotus seed protein. Under high-power microwave irradiation, sub bonds of the protein were broken, causing disaggregation and unfolding of the secondary structure, namely a decrease in the intermolecular aggregate structure and increase in the random coil structure, making the protein bonds susceptible to papain in the enzymolysis. On the other hand, a response surface methodology (RSM) was launched to investigate the influence of the enzymolysis process variables on the DH (degree of hydrolysis). The statistical analysis revealed that the optimized conditions were a protein substrate concentration of 15 g/L, pH of 5.5, enzymolysis temperature of 57 °C, papain amount of 0.5 g/L, and enzymolysis time of 45 min, for which the predicted value of the DH was 35.64%. The results indicated that a microwave also had better potential for applications in the enzymolysis of foods.

## 1. Introduction

Lotus (Nelumbo nucifera, Gaertn) is a perennial aquatic herb with a creeping rhizome that is found throughout the world [1]. Today, lotus is widely cultivated and consumed in Asia, Oceania, and America [2]. All parts of the lotus are exploitable; its parts are often used in gastronomy and health care. It has been reported that lotus contains abundant functional components, including polysaccharides, polyphenols, flavonols, procyanidins, and alkaloids [3].

Lotus seeds, which are rich in carbohydrates, proteins, amino acids, and alkaloids, are widely used as food and pharmaceutical products in India, China, and Japan [4], and its starch as other starches is used as a thickening, stabilizing, gelling, filling, and water retention agent in food and non-food applications [5]. Although the lotus seed protein is believed to be of a good nutritional value, lotus seed protein is not widely used in food applications due to the competitiveness of other traditionally more accepted pulses, such as soybean proteins. A number of drawbacks are associated with the use of soybean proteins in foods, such as their beany flavor and the presence of anti-nutritional factors such the Kunitz trypsin inhibitor (20 kDa), Bowman-Birk protease inhibitor (7.9 kDa), and soybean agglutinin (120 kDa) [6], causing the intervention of the following antibodies: antibody IgG, IgA, and IgM to soya protein. The effects of diets based upon soya protein resulted, among other things, in the inhibition of abomasal emptying, a decreased transit time of markers through the small intestine, abnormal water and salt exchange, and decreased nitrogen absorption [7], and the introduction of genetically modified organisms has roused interest in alternative vegetable protein sources, including lotus seed. Exploration of other plant proteins may meet the increasing demand of balanced nutrition in humans [8]. Therefore, increasing attention is being placed on the extraction and characterization of new bioactive polysaccharides from edible fruits, which may be applied to functional foods and medicine [9]. However, the use of bioactive components of lotus seed is limited by the low availability of its functional properties when it is consumed directly.

Enzymatic hydrolysis of proteins can be used to release bioactive fractions [10], and partial enzymolysis without affecting the nutritional value of the food is the major process employed to improve the functional properties of dietary proteins. These functional properties of protein hydrolysates are often determined by the degree of hydrolysis (DH).

We have already worked on this topic as Pan et al (2016) [11]. However, this previous report did not address the aspect of structural changes and quantified the irradiation powers under which these changes occurred. 

Enzymolysis of proteins after microwave irradiation pretreatment is a new technique used for accelerating protein hydrolysis and improving their functional properties due to influencing the proteins in a short time. Microwave-assisted procedures using enzyme and/or various acid mixtures have been widely employed to cause changes in organic matrices of biological samples [12]. The biological variations that can arise on the proteins after pre-treatment with a microwave are a function of the intensity of the field of treatment. Microwave heating associated with non-thermal effects could be responsible for these structural modifications of the proteins, resulting in a change in the secondary and tertiary structure of proteins after absorbing microwave energy [13]. Microwaves offer a clean, rapid, convenient, and cost effective method for heating that can cause an athermal effect by polarizing macromolecules, resulting in alignment with electromagnetic field poles that may cause the breakage of hydrogen bonds [14]. Several recent reports have focused on the application of microwaves as a preferred technique for accelerating the hydrolysis of proteins. For example, microwave-assisted extraction (MAE) uses microwaves’ power to stimulate the molecular motion and spin of liquids’ molecules with a constant dipole [15]. Microwave pretreatments can increase the DH and solubility of proteins [16], ultrasonic/microwave assisted treatment on the properties of soy protein isolate/microcrystalline wheat-bran cellulose film [17], efficient production of glucose by microwave-assisted acid hydrolysis of cellulose hydrogel [18], and evaluation of bromine and iodine content of milk with proteins combining digestion by microwave-induced combustion and inductively coupled plasma mass spectrometry (ICP-MS) determination [19]. MAE has several advantages over conventional extraction techniques, including a modified yield, reduced extraction time, and lower solvent cost, as well as a high level of automation [20,21]. Additionally, it is an environmentally friendly process with production efficiency and contributes to the preservation of the environment by reducing the use of water and solvents, the evacuation of wastewater, fossil energy, and the production of dangerous substances [22]. However, despite the advantages of the proposed approach, the application of a microwave to lotus seed proteins is barely depicted explicitly in the literature.

This study reports that microwave-assisted technology coupled with papain could improve the yield of the bioactive peptides production from lotus seed. With the use of a microwave, several aspects not developed in depth in our previous article will be the subject of investment here, in addition to the reassertion of certain experiments in order to confirm them. A deduction will be drawn from the efficiency of the use of a microwave compared to the thermal treatment of our previous study [11]. 

To probe lotus seed protein enzymolysis for bioactive peptides production, the effects of microwave pretreatment parameters included microwave power and irradiation time on the DH were ascertained. The ATR-FTIR spectroscopic technique was used in order to better understand the capabilities of a microwave in terms of the lotus seed protein in the pretreatment process. Finally, the response surface methodology (RSM) was used to optimize the enzymolysis parameters (temperature, pH, protein substrate concentration, and papain amount). 

## 2. Experimental

### 2.1. Materials

Lotus seeds without integument and embryos were purchased on a wholesale market in Xiangtan (Xiangtan, China). Papain (EC3.4.22.2, ≥99%) was purchased from Sigma-Aldrich Company Ltd (Shanghai, China). All other reagents used in the experiment were of analytical grade and used without further purification. According to the previous description in the literature by Zeng et al (2013) [23] and Pan et al. (2016) [11], the lotus seed protein (albumin + globulin) was extracted in 0.1 mol⋅L^−1^ NaCl solution with a flour/solvent ratio of 1:10 w/v at 40 °C for 1 h (constant magnetic stirring). The slurry was centrifuged at room temperature (4000 rpm, 20 min), and the supernatant was adjusted to pH 4.2 by adding 0.1 mol/L HCl solution. Next, the residue was centrifuged and the liquid was decanted. The product was washed three times and freeze dried. The protein powder contained approximately 88.9% protein content, 1.1% fat, 1.8% total sugar content, and 2.9% moisture content. Before the experiments, the lotus seed was ground and milled to pass through a 100 mesh sieve to produce the whole flour.

### 2.2. Microwave Pretreatments

The protein powder was prepared in a solution containing a 35.0 g/L protein concentration. The solution was stirred for 30 min and then equilibrated in an incubator at 40 °C for 1 h, after which it was cooled to room temperature. The solution was subjected to microwave energy (XH-800AE, Beijing Xiang Hu Science and Technology Co. Ltd., Beijing, China) at a specific power (500~900 W) for 15~210 s. The microwave pretreated protein solution was used as a substrate solution in enzymolysis tests. 

### 2.3. Enzymolysis of the Pretreated Protein

The enzymolysis study was performed according to that of Pan et al (2016) [11]. The protein substrate solution was from the pretreated protein solution. This stock solution was diluted to obtain varying concentrations between 5.0 and 35.0 g/L, respectively. Based on the experimental plan design, different concentrations of papain (0.2–0.8 g/L) were added into the pretreated lotus seed protein suspension with a specific concentration (2–34 g/L), respectively. The enzymolysis was performed by employing a shake flask method with a 150 mL flask in a water bath of a controlled temperature and the reaction was quenched by heating treatment at 100 °C for 10 min. The hydrolysate (supernatant) was clarified by centrifuging at 4000 rpm for 15 min to remove insoluble substrate fragments and residual enzyme. Analysis of the hydrolysate was then carried out by determining the degree of hydrolysis (DH) through measuring the nitrogen content in solution, by applying the 10% trichloroacetic acid method, as discussed by Kim et al (2015) [24], and was defined as follows:

DH (%) = 100× *N*1/*N*2 (1)

where *N*1 was the soluble nitrogen in the hydrolysate and *N*2 was the total nitrogen content in the protein powder.

The response surface methodology (RSM) is a powerful statistical tool. It is often applied when testing multiple process variables with fewer experimental trials, and can evaluate the effects of several factors and the interactions between them at the same time. RSM possesses capabilities in estimating and predicting the effect of several different quantities at the same time with respect to the fact that measuring these properties in the laboratory is time-consuming and costly, and includes the existence of regular experimental errors [25]. RSM was used to evaluate the effect of enzymolysis temperature, initial pH, protein substrate concentration, and papain amount on the DH in the enzymolysis of the pretreated protein. The central composite design (CCD) was used to design the experiment and optimize one response variable, namely the DH (*Y*), from the enzymolysis of lotus seed protein. Each independent variable was coded at five levels between −2 and +2, where the variables of the protein substrate concentration (S), pH (P), enzymolysis temperature (T), and papain amount (E) were changed in the ranges shown in Table 1. 

Thirty experiments were augmented with three replications at the design center to evaluate the pure error and were carried out in a randomized order, as required in many design procedures. After the reaction, the response *Y* was measured. The statistical software package Design Expert software (version 8.0.6) was used for regression analysis of the experimental data and to plot the response surface. The model generated during RSM implementation was validated by conducting experiment on a given optimal setting. The second-order polynomial model was applied to predict the response variable (*Y*), as shown below:

(2) $$Y=\beta_{0}+\sum_{i=1}^{4}\beta_{i}X_{i}+\sum_{i=1}^{4}\beta_{ii}X_{i}^{2}+\sum_{i=1}^{3}\sum_{j=i+1}^{4}\beta_{ij}X_{i}X_{j}$$

where *Y* was the response value (degree of hydrolysis); *β*_0_, *β_i_*, *β_ii_*, and *β_ij_* were the regression coefficients for interception, linear, quadratic, and interaction terms, respectively; and *X_i_* and *X_j_* were the independent variables. 

### 2.4. ATR-FTIR Spectroscopy

The pretreated protein substrate solution was used for attenuated total reflectance Fourier transform infrared spectroscopy (ATR-FTIR) measurements. ATR-FTIR spectra were recorded on a PE Spectrum One B instrument equipped with a horizontal ATR crystal (ZnSe, 45°), working under vacuum to avoid intense spectral components due to the existence of CO_2_ and H_2_O in the atmosphere. Background was subtracted using the Opus software. Curve fitting was then performed using Origin 8.0 software and Peak Fit v4.12. Fourier self-deconvolution was conducted on the average spectra for the amide I band (1700~1600 cm^−1^), using a resolution enhancement factor of 1.8 and full height band width of 13 cm^−1^. Information on the number and location of components was obtained through Self-deconvolution. Gaussian curve-fitting was then performed using Origin 8.0 software (Savitsky-Golay as a derivative operation). The minimum value of the spectrum of the second derivative was the criterion of choice of the best adjustment procedure.

### 2.5. Statistical Analysis

Each standard and sample were measured in triplicate. The mean and standard deviation (n = 3) were calculated. The data were statistically analyzed at the significance level of *p* < 0.05 using Levene’s test for homogeneity and Duncan’s multiple range test with SAS version 9.2 (English) to determine if there were differences between treatments.

## 3. Results and Discussion

### 3.1. Microwave Pretreatment

Peptide products of the lotus seed protein after the microwave pretreatment were prepared using papain under the condition of an enzymolysis temperature of 50 °C, pH of 5.5, protein substrate concentration of 12 g/L, and papain amount of 0.5 g/L. It demonstrated that microwave pretreatment could be used to reinforce the efficiency of the enzymolysis in the preliminary tests [26]. A microwave causes water evaporation in the plant cells and increases the pressure in the internal environment, leading to cell material decomposition and membrane disruption [27], and thus disintegration in the cell which facilitates the process of enzymolysis of its proteins. It can cause different biological and chemical effects, depending on the strength and duration of exposure during the pretreatment process [28]. 

#### 3.1.1. Influence of Microwave Power 

**Figure 1:**
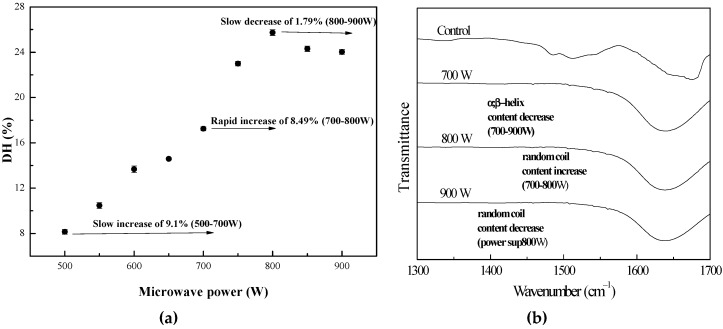
Influence of microwave irradiation power on (**a**) DH and (**b**) the conformation of the lotus protein during enzyme hydrolysis.

The changes in the DH of the pretreated protein in the enzymolysis by papain were examined under different microwave powers (500 W to 900 W) for 120 s, and the results are shown in Figure 1. Pretreatments of the lotus seed protein with microwave irradiation before the enzymolysis had different effects on the DH during the enzymolysis. As shown, the apparent DH of the lotus seed protein sample was increased significantly when compared to the control (at 500 to 900 W) (*p* < 0.05). However, the increase was not the same for all microwave irradiation powers. It was also found that when the microwave irradiation power was between 700–800 W, the DH increased more than other intervals of microwave irradiation powers. Increasing the microwave power from 500 to 800 W gave a significant increase in the DH ranging from 8.2 to 25.8%, and the DH then decreased to 24.0% at above 800 W. It was possible that the change in the DH might be due to the alteration of the secondary structure changes of the lotus seed protein [29] and that microwave pretreatment caused significant structural changes in the seeds’ tissues [27].

Changes in the secondary structure of proteins have often been investigated using ATR-FTIR spectroscopy. The amide I band, observed between 1700 and 1600 cm^−1^, is the most useful for spectroscopic analysis of the secondary structure of proteins. The amide band I peptide bonds in proteins are most commonly used in vibration analysis, in addition to having a high susceptibility to secondary structure changes. The secondary structure of the proteins influences these vibrations, which results in the folding of the proteins of the hydrogen bonds between the peptide bonds.

Amide I band analysis might, therefore, be used to study differences in the secondary structure of the proteins [30,31]. The conformational changes in the pretreated proteins were evaluated by ATR-FTIR, and the original ATR-FTIR spectra of the individual pretreated protein sample in the 1700~1300 cm^−1^ region were investigated in Figure 1. The second derivative compositions comprising the intermolecular aggregate, *α*-helix, *β*-sheet, *β*-turn, and random coil were calculated in the Amide I region (1700~1600 cm^−1^), and the components’ peaks, locations, and percentage are summarized in Table 2. The amide I component at 1655 cm^−1^ was assigned to *α*-helix modes, and the ones at 1625 and 1640 cm^−1^ to *β*-sheet modes. The component at around 1615 was attributed to the intermolecular aggregate, and the 1668 and 1688 cm^−1^ components could be attributed to vibration modes created in turn (*β*-turn) in the *β*-sheet structures. The existence of an unordered conformation (random coil) was observed at about 1645 cm^−1^ [32,33]. Microwave irradiation power had an effect on the secondary structure of the lotus seed protein, where the contents of the secondary structure of the protein samples changed to some extent though the number of peaks displayed no significant changes. From Table 2, it could be observed that the corresponding content of the protein secondary structure before and after being exposed to the microwave field varied significantly. The *α*-helix content of the pretreated protein was remarkably lower than that of the native l protein (control), and dramatically decreased with an increase in the microwave power. At the same time, the *β*-sheet content also decreased with an increase in the power, and was lower than the control. On the other hand, the random coil content increased with aggrandizement of the power from 700 to 800 W and then declined, but instead, the intermolecular aggregate content reduced with the advance of the power and then increased at above 800 W. The random coil content of the pretreated protein was higher than the control, but in contrast, the intermolecular aggregate content was lower than the control.

The results described above supported the view that a microwave can bring about structural protein rearrangement with an increase of the ordered structures [34]. A microwave is an electromagnetic wave and the heating of proteins by microwave power was rapid and uniform throughout the protein. Under high-power microwave irradiation, non-covalent bonds and the disulfide bonds in lotus seed protein molecules were broken, causing the disaggregation and unfolding of proteins, which facilitated more enzyme-cutting sites of the lotus seed proteins exposure to papain [13,35]. The DH enhanced with an increase of the random coil structure and reduction of the intermolecular aggregate structure. Therefore, it made papain more readily accessible for reaction and thus resulted in the increase of the DH. Due to the change in the conformation of the protein molecules, such as the decrease in the intermolecular aggregate structure, and *α*-helix and *β*-sheet structures and the increase in the random coil structure, the probability of the unfolding of protein molecules was enhanced, causing the DH to increase gradually. However, excessively high power (>800 W) caused the formation of new protein aggregates, although less *α*-helix and *β*-sheet structures than the pretreated protein at 800 W were found, and thus led to the decrease of the DH. 

#### 3.1.2. Influence of Microwave Time

**Figure 2:**
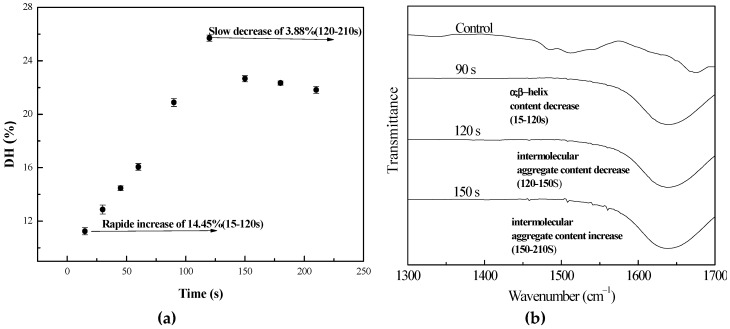
Influence of microwave irradiation time on (**a**) DH and (**b**) the conformation of the lotus protein during enzyme hydrolysis.

The effect of the microwave pretreatment time on the DH under 800W microwave power after 15 to 210 s is shown in Figure 2. The variation of DH varied significantly (*p* < 0.05) from one interval time to the other. The DH values as a function of exposure time ranged from 11.25 to 25.7%, and the results showed that the DH of the lotus seed proteins increased rapidly in the first 120 s, before the DH then declined. Microwave pretreatment gave the highest DH (25.7%) in the enzymolysis after 120 s. The conformational changes in the ATR-FTIR spectra of the pretreated lotus seed proteins are shown in Figure 2 and Table 2. The changes of the secondary structures under different microwave powers and times exhibited a similar trend in that the *α*-helix and *β*-sheet contents of the proteins decreased with the extending of pretreatment time, but the random coil content increased in 120 s and then dropped, as confirmed by the study by [36]. Meanwhile, the intermolecular aggregate decreased in 120 s and then increased. The results confirmed the presumption that a decrease in the intermolecular aggregate structure and an increase in the random coil structure induced the disintegration and unrolling of protein molecules, causing the DH to increase in accordance with Ni et al’s (2015) [29] report.

In conclusion, a microwave has the ability to break down aggregates of the lotus seed protein molecules, making the protein bonds susceptible to papain in the enzymolysis. However, this greatly depends on the power and length of microwave pretreatment, and the best condition of microwave pretreatment is 800 W for 120 s in 35.0 g/L lotus seed protein solution. This pretreatment condition was used for the following enzymolysis experiments.

### 3.2. Enzymolysis

#### 3.2.1. Central composite design (CCD) analysis

This study was carried out under CCD, which is the most used matrix of the response surface methodology (RSM). CCD was applied because it helps to optimize effective parameters and analyze interactions between parameters, with a full capability for orthogonal blocking and better prediction quality; however, it requires a greater number of experiments than Box-Behnken design [37]. The center point in the conception was repeated five times for the estimation of experimental errors. The data representing the independent variables and the responses obtained during each multivariate experiment are listed in Table 3.

Statistical analysis of variance (ANOVA) was used to investigate not only the significance of the model and fitness, but also the interaction effects between independent variables and the effects of the individual variables on the response (Table 4). On the basis of the investigation carried out with the use of ANOVA, it was deduced that the model was highly significant with a *p*-value of less than 0.0001 to predict the response values. As regards significant coefficients, the variables S (protein substrate concentration) and P (pH) were highly significant factors (*p* < 0.0001), and T (enzymolysis temperature) was a significant term (*p* < 0.05), but E (papain amount) was insignificant. The quadratic terms of ST and PT were very significant terms (*p* < 0.0001), but SP, SE, and TE were insignificant (*p* ≥ 0.05). The quadratic terms of S^2^, P2, and T^2^ were highly significant terms (*p* < 0.0001), and E^2^ was a significant term (*p* < 0.05) [38]. The elimination of the insignificant terms could improve the regression model, and the quadratic model was given as:

Y = 32.12 − 4.18S + 3.71P + 0.92T − 1.53ST − 1.31PT − 3.12S^2^ − 1.40P^2^ − 1.27T^2^ − 0.80E^2^ (3)

Evaluation of the determination coefficient (R^2^) and the regression equation made it possible to test the suitability of the model. The determination coefficient value (R^2^ = 0.9884) in this study implied that the model terms were statistically significant. The correlation coefficients between the predicted data by the model and the achieved experimental data revealed the desirability of the suggested model in the prediction of the selected responses [39]. The predicted R^2^ is the parameter that measures the quality (how good or bad) of the model; here, it gave predicted response values within 0.0252 of the adjusted R^2^ that verified experimental data and model accuracy. Furthermore, the predicted R^2^ and adjusted R^2^ were both close to 1, which indicated the adequacy of the model [40]. Adequate precision is a measure of the signal to noise ratio, which compared the range of the predicted values at the design points to the average prediction error. An adequate precision value was found to be 37.846 (greater than 4), indicating that the model was sufficient for discrimination. On the other hand, a relatively lower value of the coefficient of variation (CV 3.44%) indicated the good precision and reliability of the experiments [41]. Comparing residual error to pure error, the non-significant value of lack of fitness (*p* > 0.05) also confirmed in the studied case that the quadratic model was valid. 

#### 3.2.2. Interactions between the Variables

**Figure 3:**
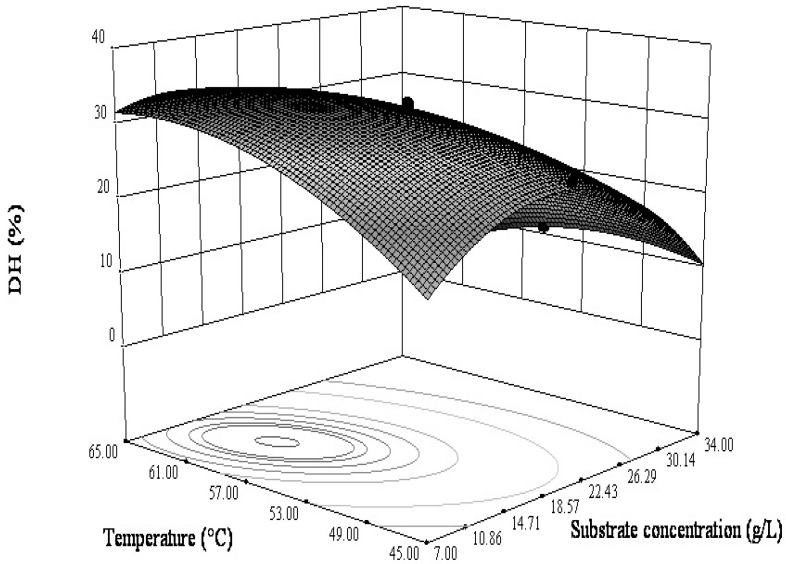
The combined effect of protein concentration and hydrolysis temperature on the DH at constant pH (5.0) and papain amount (0.5 g/L).

**Figure 4:**
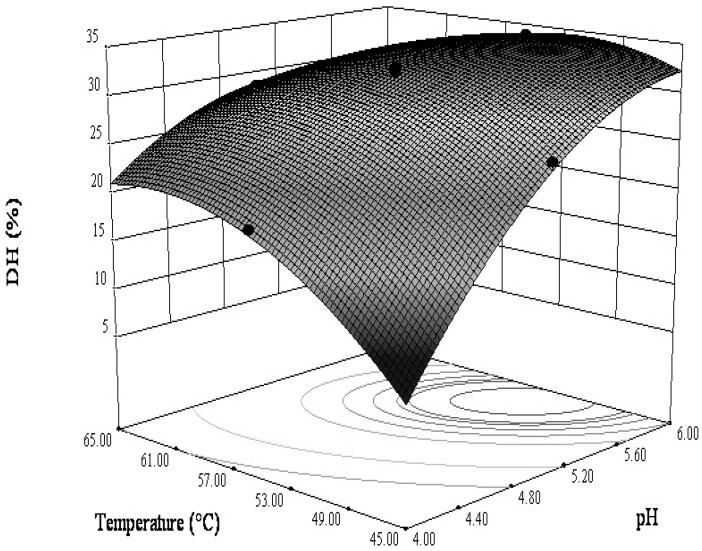
The combined effect of constant pH and hydrolysis temperature on the DH at protein concentration (20 g/L) and papain amount (0.5 g/L).

The model suggested that highly significant interactions were noted between protein substrate concentration and enzymolysis temperature, as well as between pH and enzymolysis temperature. Accordingly, it becomes very important to further characterize interactions in the range of process variables. The 3D response surfaces and 2D contour plots for the DH optimization are shown in Figure 3 and Figure 4, with each representation showing the individual and/or interactive effect of two variables on the DH, while the other two variables are kept at the zero level. Figure 3 represented the combined effect of enzymolysis temperature and protein substrate concentration on the DH. The elliptical nature of the contour plot indicated that the interaction of the enzymolysis temperature and substrate concentration was considerable in the enzymolysis. The temperature had a linear effect on the response from 45 to 59.5 °C, where DH increased with temperature and then reached equilibrium as the temperature continued to rise. However, the substrate concentration demonstrated a quadratic effect on the response, where the DH increased up to about 13.76 g/L, followed by a decline with an increase in the substrate concentration. 

The combined effect of the enzymolysis temperature and pH on the DH is shown in Figure 4. The contour line with an elliptical shape demonstrated that the combined effect of the enzymolysis temperature and pH on the DH was significant. Both the temperature and the pH of enzymolysis exerted effects on DH when the temperature and pH were respectively below 52.9 °C and 5.76, and then reached equilibrium with increasing temperature and pH. Though the current situation could be more complex, here, an attempt of enzymolysis optimization and description of the process by RSM is proposed.

#### 3.2.3. Optimization Analysis

One of the objectives of this study was to get the optimum operative parameters to maximize the DH from the expanded mathematical model equation. In total, 35.64% of the maximum value of DH predicted by the model was obtained when the protein substrate concentration, pH, enzymolysis temperature, and amount of papain were 15.10 g/L, 5.5, 56.78 °C, and 0.53 g/L, respectively. Confirmation and validation of the chosen model was done by determining the optimal conditions of the enzymolysis reaction after further experiments. In conclusion, a protein concentration of 15 g/L, a pH of 5.5, an enzymolysis temperature of 57 °C, and an amount of papain of 0.5 g/L during 45 min represented the conditions to be met for reaching the maximum DH. According to the results, the experimental DH (34.72%) and the predicted DH (35.64%) did not differ significantly, which confirmed the effectiveness of the predicted model. With an improvement in the DH efficiency of about 8% (34.72% > 26.84%), it could be deduced that pre-treatment with a microwave is much better than that used in our previous study. Therefore, it could be stated that the application of these intelligent systems might determine the intended characteristics quickly and accurately, beside saving the cost and time [25].

## 4. Conclusions

Pretreatment of the lotus seed protein by a microwave before enzymolysis enhanced the enzymolysis of the protein, and the DH increased to the highest value (25.8%) at 800 W for 120 s. The microwave could break sub bonds in the molecule and cause disaggregation and unfolding, facilitating the protein enzymolysis. The enzymolysis was optimized using RSM to elevate the DH, and was significantly affected by the protein concentration, pH, and enzymolysis temperature. The optimum conditions of a protein concentration of 15 g/L, pH of 5.5, enzymolysis temperature of 57 °C, papain amount of 0.5 g/L, and enzymolysis time of 45 min were obtained with the predicted highest DH of 35.64%.

## Figures and Tables

**Table 1:** Experimental range and levels of the independent variables.

Independent Variables	Symbols	Units	Code Levels				
			−2	−1	0	+1	+2
Substrate concentration	S	g/L	7.0	13.0	20.0	27.0	34.0
pH			4.0	4.5	5.0	5.5	6.0
Hydrolysis temperature	T	°C	45	50	55	60	65
Papain amount	M	g/L	0.3	0.4	0.5	0.6	0.7

**Table 2:** Areas and assignments of the secondary structures in amide I infrared bands from the pretreated proteins.

Assignment	Peak Centers (cm^−1^)	Control	Time (s)			Power (W)		
			90	120	150	700	800	900
Intermolecular aggregate	1615	12.97	10.71	6.00	9.24	13.89	5.23	11.88
*β*-Sheet	1625	13.00	24.25	26.21	34.45	29.57	24.51	26.43
	1640							
*α*-Helix	1655	27.2	22.51	13.70	11.05	19.97	18.02	17.12
Random	1645	14.63	19.02	29.17	17.95	15.87	28.57	15.23
*β*-Turn	1668	32.19	23.49	24.93	27.31	20.68	23.66	29.32
	1688							

**Table 3:** Central composite design matrix and its response and predicted value.

Run	Experimental Variables				Response *Y* (%)	
	S (g/L)	P	T (°C)	E (g/L)	Expt.	Predicted
1	13	4.5	50	0.4	22.25	23.13
2	27	4.5	50	0.4	17.74	17.75
3	13	5.5	50	0.4	31.36	32.21
4	27	5.5	50	0.4	29.52	28.77
5	13	4.5	60	0.4	29.43	29.15
6	27	4.5	60	0.4	16.30	17.65
7	13	5.5	60	0.4	33.72	32.99
8	27	5.5	60	0.4	23.95	23.43
9	13	4.5	50	0.6	22.57	22.30
10	27	4.5	50	0.6	14.38	15.13
11	13	5.5	50	0.6	31.47	31.38
12	27	5.5	50	0.6	25.41	26.15
13	13	4.5	60	0.6	30.56	31.33
14	27	4.5	60	0.6	19.68	18.04
15	13	5.5	60	0.6	34.72	35.17
16	27	5.5	60	0.6	23.43	23.81
17	6	5.0	55	0.5	28.55	27.99
18	34	5.0	55	0.5	11.19	11.27
19	20	4.0	55	0.5	19.62	19.07
20	20	6.0	55	0.5	33.85	33.93
21	20	5.0	45	0.5	26.02	25.2
22	20	5.0	65	0.5	28.54	28.89
23	20	5.0	55	0.3	29.31	29.14
24	20	5.0	55	0.7	29.01	28.70
25	20	5.0	55	0.5	32.51	32.12
26	20	5.0	55	0.5	32.71	32.12
27	20	5.0	55	0.5	32.64	32.12
28	20	5.0	55	0.5	32.28	32.12
29	20	5.0	55	0.5	31.69	32.12
30	20	5.0	55	0.5	30.87	32.12

**Table 4:** ANOVA analysis for response surface quadratic model (*α* = 0.05).

Source	Sum of Squares	DF	Mean Square	F	*p*-Value
Model	1160.19	13	89.25	104.43	<0.0001
S	419.92	1	419.92	491.39	<0.0001
P	331.01	1	331.01	387.34	<0.0001
T	20.41	1	20.41	23.88	0.0002
E	0.29	1	0.29	0.34	0.5666
SP	3.75	1	3.75	4.39	0.0523
ST	37.42	1	37.42	43.79	<0.0001
SE	3.21	1	3.21	3.76	0.0703
PT	27.48	1	27.48	32.16	<0.0001
TE	9.05	1	9.05	10.58	0.0050
S^2^	267.16	1	267.16	312.63	<0.0001
P^2^	54.12	1	54.12	63.33	<0.0001
T^2^	44.13	1	44.13	51.64	<0.0001
E^2^	17.49	1	17.49	20.46	0.0003
Residual	13.67	16	0.85	-	-
Lack of Fit	11.13	11	1.01	1.99	0.2315
Pure Error	2.54	5	0.51	-	-
Cor Total	1173.86	29	-	-	-
R^2^	0.9884				
Adjusted R^2^	0.9789				
Predicted R^2^	0.9537				
Adeq precision	37.846				
CV	3.44%				
